# Neoadjuvant Chemotherapy Prior to Trimodality Therapy for Muscle-invasive Bladder Cancer: A Systematic Review and Meta-analysis

**DOI:** 10.1016/j.euros.2025.11.005

**Published:** 2025-12-04

**Authors:** Keiichiro Miyajima, Akihiro Matsukawa, Takafumi Yanagisawa, Marcin Miszczyk, Navid Roessler, Shota Inoue, Abdulrahman S. Alqahtani, Ahmed R. Alfarhan, Fumihiko Urabe, Keiichiro Mori, Pierre I. Karakiewicz, Takahiro Kimura, Shahrokh F. Shariat

**Affiliations:** a Department of Urology, Comprehensive Cancer Center, Medical University of Vienna, Vienna, Austria; b Department of Urology, The Jikei University School of Medicine, Tokyo, Japan; c Division of Anatomy, Medical University of Vienna, Vienna, Austria; d Collegium Medicum – Faculty of Medicine, WSB University, Dąbrowa Górnicza, Poland; e Department of Urology, University Medical Center Hamburg-Eppendorf, Hamburg, Germany; f Department of Urology, Okayama University Graduate School of Medicine, Dentistry and Pharmaceutical Sciences, Okayama, Japan; g Department of Urology, Ministry of Health, Second Health Cluster, Riyadh, Kingdom of Saudi Arabia; h Department of Urology, Prince Saud Bin Jalawi Hospital, Al Ahsa Health Cluster, Al Ahsa, Kingdom of Saudi Arabia; i Cancer Prognostics and Health Outcomes Unit, Division of Urology, University of Montréal Health Center, Montréal, Québec, Canada; j Department of Urology, University of Texas Southwestern Medical Center, Dallas, TX, USA; k Hourani Center for Applied Scientific Research, Al-Ahliyya Amman University, Amman, Jordan; l Department of Urology, Weill Cornell Medical College, New York, NY, USA; m Department of Urology, Semmelweis University, Budapest, Hungary; n Karl Landsteiner Institute of Urology and Andrology, Vienna, Austria

**Keywords:** Bladder preservation, Meta-analysis, Muscle-invasive bladder cancer, Neoadjuvant chemotherapy, Trimodality therapy

## Abstract

**Background and objective:**

Neoadjuvant chemotherapy (NACT) before radical cystectomy improves outcomes in muscle-invasive bladder cancer (MIBC), but its value before trimodality therapy (TMT; maximal transurethral resection of the bladder tumor plus concurrent chemoradiation) is uncertain. This review aims to evaluate whether the addition of NACT to TMT is associated with improved survival in patients with MIBC.

**Methods:**

We systematically searched the MEDLINE, Embase, and Web of Science databases (February 2025). Eligible studies reported adjusted estimates for overall (OS), cancer-specific (CSS), or disease-free (DFS)/recurrence-free survival in patients undergoing TMT, comparing those who received versus those who did not receive NACT. Random-effect meta-analyses pooled multivariable hazard ratios (HRs). The risk of bias was assessed with ROBINS-I (PROSPERO registration: CRD420251120157).

**Key findings and limitations:**

Fourteen studies comprising 4112 patients met the inclusion criteria. However, only two to three studies contributed adjusted HRs for each oncological outcome regarding the use of NACT or the response to NACT. Gemcitabine-cisplatin was the most common NACT regimen (reported in ten studies). Compared with no NACT, NACT was not associated with a statistically significant improvement in OS (HR 0.93, 95% confidence interval [CI] 0.78–1.12, *p* = 0.40; three studies, *n* = 3500) or CSS (HR 0.76, 95% CI 0.51–1.12; *p* = 0.16; two studies, *n* = 934). Among patients who received NACT, clinical/pathological response was associated with better outcomes (OS: HR 0.26, 95% CI 0.08–0.86, *p* = 0.03; two studies, *n* = 74; DFS: HR 0.42, 95% CI 0.25–0.70, *p* < 0.001; two studies, *n* = 116). Overall certainty is limited by nonrandomized design, heterogeneity in patient selection, TMT protocols and response definitions, and the modest number of adjusted studies.

**Conclusions and clinical implications:**

Across adjusted observational data, addition of NACT before TMT was not associated with a survival benefit. However, patients who respond to NACT experience a substantially better prognosis. Prospective trials testing modern neoadjuvant strategies—particularly immunotherapy- and targeted therapy-based regimens—within the standardized TMT protocols are warranted.

**Patient summary:**

We reviewed studies in which chemotherapy was given before a bladder-sparing approach, called trimodality therapy (tumor removal plus chemoradiation). Overall, this treatment approach did not improve survival, although patients who responded to the pretreatment did better. Newer drugs, including immunotherapy, should be tested in this setting.

## Take Home Message

Neoadjuvant chemotherapy before trimodality therapy is not associated with a survival benefit overall; responders fare better. Prospective, biomarker-guided trials of immunotherapy/targeted neoadjuvant strategies within the trimodality therapy are warranted.

## 1 Introduction

Radical cystectomy (RC) with perioperative systemic therapy remains the standard of care for nonmetastatic muscle-invasive bladder cancer (MIBC) [1], [2], [3], [4]. Although RC provides durable local control, urinary diversion can impair quality of life markedly [5], [6], prompting interest in bladder-preserving strategies. Trimodality therapy (TMT)—maximal transurethral resection of the bladder tumor (TURBT) followed by radiotherapy with concurrent chemotherapy—has emerged as an alternative for carefully selected patients [4]. Contemporary series suggest that TMT may achieve survival outcomes comparable with RC, while maintaining quality of life through bladder preservation [7], [8], [9]. However, TMT protocols are not standardized and practice varies substantially across institutions [10], [11].

Neoadjuvant chemotherapy (NACT) improves oncological outcomes before RC [3], [12], but its role in the TMT setting remains uncertain [7]. We therefore conducted a systematic review and meta-analysis to assess whether addition of NACT to TMT improves overall survival (OS; primary endpoint), with cancer-specific (CSS) and disease-free (DFS) survival as the prespecified secondary endpoints.

## 2 Methods

The protocol was registered in PROSPERO (CRD420251120157).

### 2.1 Search strategy

This systematic review and meta-analysis followed the Preferred Reporting Items for Systematic Reviews and Meta-analyses (PRISMA) 2020 and Meta-analysis Of Observational Studies in Epidemiology (MOOSE) guidelines for reporting (Supplementary Table 2) [13], [14], and methodological quality was assessed using AMSTAR-2 (Supplementary material) [15]. In February 2025, we searched MEDLINE (PubMed), Embase, and Web of Science Core Collection for studies evaluating the association of NACT with oncological outcomes in patients with MIBC treated with TMT. Full database-specific strategies are provided in the Supplementary material.

The primary outcome was OS; CSS and DFS/recurrence-free survival (DFS/RFS) were secondary outcomes. Two reviewers (K.M. and S.I.) independently screened titles/abstracts and then full texts; disagreements were resolved by discussion with additional coauthors.

### 2.2 Inclusion and exclusion criteria

Using the PICOS framework [16]: population—adults with nonmetastatic MIBC undergoing TMT (maximal TURBT plus external-beam radiotherapy with concurrent radiosensitizing chemotherapy); intervention—NACT before TMT; comparator—TMT without NACT; outcomes—OS, CSS, and DFS/RFS; study design—observational or randomized studies.

We included studies that accounted for confounding (eg, multivariable regression or propensity methods). We excluded studies that (1) reported only unadjusted estimates, (2) did not present original patient data (reviews, editorials, and letters), (3) were not in English, or (4) evaluated nonstandard bladder-sparing approaches (eg, partial cystectomy and TURBT plus radiotherapy without concurrent chemotherapy). Reference lists of eligible articles were hand searched for additional studies.

### 2.3 Data extraction

Two authors (K.M. and S.I.) independently extracted data using a standardized form: first author, year, country and recruitment period, sample size, age, T stage, NACT regimen and cycles, total radiation dose (Gy) and concurrent chemotherapy, follow-up, salvage cystectomy rate, and oncological outcomes (OS, CSS, DFS/RFS, metastasis-free survival, and progression-free survival). We extracted adjusted hazard ratios (HRs) with 95% confidence intervals (CIs) from the most fully adjusted models. Only studies that reported adjusted HRs for oncological outcomes were eligible for inclusion in the quantitative synthesis. For each study, we extracted the number of observed events (death for OS, cancer-specific death for CSS, and recurrence or progression for DFS) separately for the NACT and no-NACT arms whenever available. When multiple publications overlapped, we used the most comprehensive or methodologically robust dataset to avoid double counting. Discrepancies were resolved by consensus with coauthors.

### 2.4 Risk of bias assessment

As all the included studies were nonrandomized, the risk of bias was assessed with ROBINS-I [17]. Two authors (K.M. and S.I.) performed assessments independently. Any disagreements between the two independent reviewers were resolved through discussion, and a third reviewer was consulted when necessary.

### 2.5 Statistical analysis

Given the expected clinical and methodological heterogeneity, we used a random-effect inverse-variance meta-analysis (restricted maximum likelihood for τ^2^) on log HRs and standard errors, back-transforming results for presentation. We analyzed the following: (1) the association of NACT use (yes vs no) with OS, CSS, and DFS/RFS as the primary comparison, and (2) among NACT recipients, the association of a response (complete response [CR] vs non-CR, defined as any response other than a CR: partial response, stable disease, or progression) with outcomes as a separate responder analysis. As responder analyses were restricted to patients who received NACT, these populations are distinct from the cohorts included in the primary comparison and therefore do not directly estimate the effect of NACT versus no NACT.

We presented forest plots with pooled HRs and 95% CIs. Heterogeneity was evaluated with Cochran’s Q, I^2^, and τ^2^; where appropriate, we report 95% prediction intervals. When ten or more studies were available, we explored small-study effects by funnel plots and Egger’s test. We planned to perform prespecified sensitivity analyses (eg, leave-one-out analysis) or subgroup analyses (eg, according to the NACT regimen, TMT radiosensitizer, or study period) to explore potential sources of variability if significant heterogeneity was observed (Q test, *p* < 0.05). All analyses were conducted in R (version 4.5.0; R Foundation for Statistical Computing, Vienna, Austria) using the meta package. Two-sided *p* < 0.05 was considered statistically significant. The certainty of evidence for each oncological outcome was assessed using the Grading of Recommendations Assessment, Development and Evaluation (GRADE) tool [18]. No ethics approval was required for this aggregate data review.

## 3 Results

### 3.1 Study selection and characteristics

**Fig. 1:**
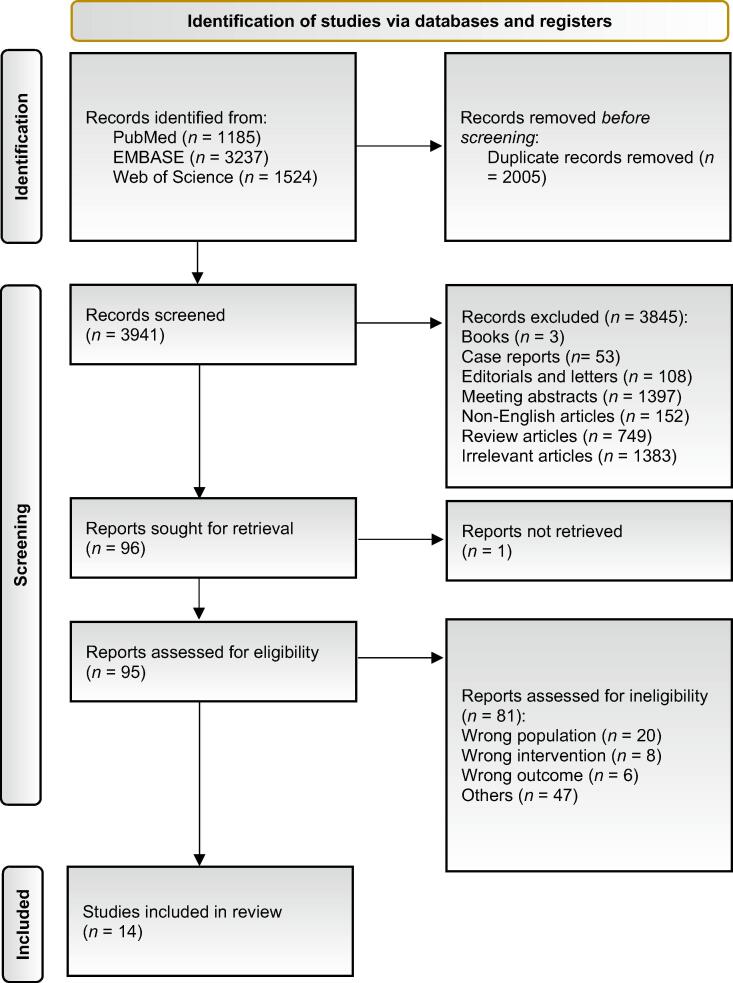
PRISMA 2020 flow diagram of study selection for the systematic review and meta-analysis. Fourteen studies (*n* = 4112) met the inclusion criteria after the screening and eligibility assessment. PRISMA = Preferred Reporting Items for Systematic Reviews and Meta-analyses.

The PRISMA flow diagram is shown in Figure 1. Fourteen studies met the inclusion criteria: three prospective nonrandomized studies (*n* = 104) [19], [20], [21], one pooled analysis of prospective studies (*n* = 348) [10], and ten retrospective cohort studies (*n* = 3,660) [22], [23], [24], [25], [26], [27], [28], [29], [30], [31]. Baseline characteristics of the included cohorts are summarized in Table 1. Most studies reported patients with clinical stage T2-T3 disease, with a minority including T4 cases. All studies applied external beam radiotherapy (typically 60–66 Gy) with concurrent chemotherapy, most commonly weekly cisplatin or gemcitabine. Tumor location was not reported in the included studies.

**Table 1:** Patient characteristics of the included studies

Author (year)	Study design	No. of NACT cycles	No. of non-NACT cycles	Median age (range)	Male (%)	Clinical T stage (%)	NACT regimen/cycles	CRT details	Response to NACT (%)	Salvage RC in NACT (%)
Agrawal (2020) [19]	Prospective nonrandomized	30	NR	61	93	T2b: 60 T3–4: 40	3 cycles of GC (86%) or GCarbo	60 Gy RT + weekly cisplatin 35 mg/m^2^	CR: 57 PR: 26	8
Ajib (2020) [22]	Retrospective	54	70	72	69	T2: 77 T3: 17 T4: 6	GC, MVAC, CMV, or gemcitabine alone (GC most common)	64–66 Gy RT to bladder/pelvic nodes + weekly cisplatin 40 mg/m^2^	NR	22
Cho (2024) [23]	Retrospective	76	NR	67 (40–85)	83	T2: 87 T3-4a: 13	GC (84%), or nivolumab + GC	NR	cCR: 87	13
Dracham (2022) [20]	Phase 2 prospective nonrandomized	40	NR	62 (50–74)	100	T2: 45 T3: 40 T4a: 15	3 cycles of GC	66 Gy RT (46 Gy phase 1, 20 Gy phase 2) + weekly cisplatin 40 mg/m^2^	CR: 23 PR >50%: 65	14
Elsayed (2023) [21]	Prospective nonrandomized	34	NR	55 (44–67)	82	T2: 29 T3: 53 T4a: 18	3 cycles of GC	64 Gy RT (44 Gy whole pelvis + 20 Gy boost to bladder) + cisplatin	CR: 62 PR >50%: 18	12
Efstathiou (2012) [10]	Pooled analysis of prospective studies	123	225	66 (27–89)	74	T2: 54T3: 38 T4: 8	2 cycles of CMV	64.8 Gy RT + cisplatin	NR	12
George (2004) [24]	Retrospective	22	38	67(44–88)	90	T2: 43 T3: 38 T4a: 19	2–4 cycles of MVAC or CMV	Median 110 Gy RT (45 Gy pelvis + 65 Gy bladder) + weekly cisplatin/carboplatin or cisplatin/5-FU	CR: 55	18
Glover (2024) [25]	Retrospective	38	NR	74 (65–78)	92	T1: 8 T2: 87 T3: 5	NR	NR	NR	NR
Jiang (2019) [26]	Retrospective	57	NR	72 (45–87)	77	T2: 76 T3: 12 T4: 12	2–4 cycles of GC	60–66 Gy EBRT + weekly cisplatin 40 mg/m^2^	CR: 19 PR: 37	14
Kool (2024) [27]	Retrospective	102	484	NACT: 65 No NACT: 77	73	T2: 78 T3–4: 22	≥3 cycles of MVAC, ddMVAC, or GC (78%)	Conventional 64–66 Gy or hypo 50–55 Gy RT + weekly cisplatin or gemcitabine or 5-FU/MMC	NR	NR
López-Araujo (2015) [28]	Retrospective	22	NR	67 (55–71)	90	T2: 55 T3: 36 T4: 9	3–4 cycles of platinum-doublet based (GC 68%: most common)	Median 108 Gy RT (45 Gy pelvis + 63 Gy bladder) + concurrent platinum-based chemo (weekly cisplatin 30 mg/m^2^ most common)	cCR: 68	NR
Nguyen (2020) [29]	Retrospective	11	42	NR	NR	NR	4 cycles of GC (75%) or MVAC	60 Gy RT (45 Gy pelvis+15 Gy bladder or 60 Gy bladder) + cisplatin, carboplatin, or 5-FU/MMC	CR: 84	2
Royce (2021) [30]	Retrospective	462	2104	NR	NR	T2: 83 T3–4: 17	NR	Median 64.5–64.8 Gy RT	NR	NR
Thompson (2017) [31]	Retrospective	38	40	NACT: 67 No NACT: 75	NR	T2: 73 T3: 23 T4: 4	3 cycles of GC	52.5 Gy RT + weekly gemcitabine 100 mg/m^2^	cCR: 82	9

cCR = clinical complete response; CMV = cisplatin, methotrexate, and vinblastine; CR = complete response; CRT = chemoradiotherapy; ddMVAC = dose-dense methotrexate, vinblastine, doxorubicin, and cisplatin; EBRT = external-beam radiotherapy; GC = gemcitabine and cisplatin; GCarbo = gemcitabine and carboplatin; 5-FU = 5-fluorouracil; MMC = mitomycin C; MVAC = methotrexate, vinblastine, doxorubicin (Adriamycin), and cisplatin; NACT = neoadjuvant chemotherapy; NR = not reported; PR = partial response; RC = radical cystectomy; RT = radiotherapy.

### 3.2 Risk of bias and study quality

Eight studies (*n* = 3853) [10], [22], [24], [25], [27], [29], [30], [31] had a moderate risk of bias and six (*n* = 259) [19], [20], [21], [23], [26], [28] had a serious risk of bias (Supplementary Fig. 1). Of the six studies (*n* = 3302) [10], [20], [21], [23], [27], [30] included in the quantitative synthesis, three (*n* = 3152) [10], [27], [30] had a moderate and three (*n* = 150) [20], [21], [23] had a serious risk of bias. Principal concerns were related to confounding inherent to observational designs, potential selection bias, and heterogeneity in patient selection and TMT protocols. These limitations may affect between-study comparability and underscore the need for prospective validation.

### 3.3 Systematic review (descriptive synthesis)

Across 14 studies (*n* = 4112), the median follow-up ranged from 12 to 92 mo (Table 2). Four studies (*n* = 233) reported 3-yr OS rates of 62–70% [21], [22], [28], [29] and four (*n* = 149) reported 3-yr DFS rates of 50–62% [20], [21], [28], [29].

**Table 2:** Details of the included studies

Author (year)	Median follow-up (mo)	Most common regimen	Response to TMT (%)	OS/DFS/other survival metrics	Oncological events reported^a^	Toxicity
Agrawal (2020) [19]	NR	GC	CR: 81 PR: 15	NR	NR	Grade 1–2 cystitis (54%), grade 1–2 diarrhea (38%), grade 3 diarrhea (3%)
Ajib (2020) [22]	43	GC	NR	3-yr OS: NACT + TMT 83% vs TMT 80% (*p* = 0.59) Overall RFS: NACT + TMT 46% vs TMT 50% (*p* = 0.70). bRFS: NACT + TMT 55% vs TMT 69% (*p* = 0.27)	NR	NR
Cho (2024) [23]	64	GC	NR	Median DFS: 46.3 mo Median MFS not reached 5-yr DFS: 38% 5-yr MFS: 70%	NR^b^ (DFS overall: 43/76)	Generally well tolerated, low urinary tract symptoms most common late complication, grade 3–4 complications in 8 patients (11%)
Dracham (2022) [20]	43 (10–66)	GC	CR: 83	3-yr OS: 70% (CR: 89%, PR ≥50%: 73%, PR <50%/SD/PD: 40%) 3-yr DFS: 51% (CR: 86%, PR ≥50%: 40%, PR <50%/SD/PD: 27%) 3-yr MFS: CR: 88%, PR ≥50%: 51%, PR <50%: 20%	OS: 1/9 (CR) vs 14/31 (non-CR) DFS: 1/9 (CR) vs 18/31 (non-CR)	Low-grade neutropenia (15%), no grade 3+ hepatic/renal/small bowel/skin toxicity, no treatment-related sepsis or deaths
Elsayed (2023) [21]	46 (6–52)	GC	NR	3-yr OS: 71% (CR: 86%, PR ≥50%: 75%, PR <50%/SD/PD: 21%) 3-yr DFS: 65% (CR: 76%, PR >50%: 40%)	OS: 5/7 (CR) vs 6/27 (non-CR)	NR
Efstathiou (2012) [10]	92 (1–256)	CMV	NR	5-yr OS: 52% 10-yr OS: 35% 5-yr CSS: 64% 10-yr CSS: 59%	NR	6 treatment-related deaths (2%)
George (2004) [24]	48.5 (10–126)	MVAC	CR: 75	5-yr OS: 36% 5-yr CSS: 54%	NR^b^ (OS overall: 36/60, DFS overall: 28/60)	NAC stopped in 4 patients (18%) due to poor tolerance/hematological toxicity
Glover (2024) [25]	36	NR	NR	2-yr OS: 76% 2-yr PFS: 60% (full NACT: 66% vs non full NACT: 38%, *p* = 0.03) 2-yr MFS: 72%	NR	28% grade ≥2 RT toxicity
Jiang (2019) [26]	19 (5–96)	GC	NR	2-yr OS: 74% 2-yr DFS: 57% 2-yr CSS: 88%	NR^b^ (OS overall: 5/57, DFS overall: 17/57)	21% developed grade 3–4 toxicities (neutropenia/infection), no grade 3–4 renal insufficiency from NACT
Kool (2024) [27]	34	GC	CR: 77	Median OS: 108 mo (NACT) vs 60 mo (no NACT) Median CSS not reached.	NR^b^ (OS overall: 229/586, CSS overall: 143/586)	NR
López-Araujo (2015) [28]	24 (6–86)	GC	CR: 77	3-yr OS: 62% (CR to NACT: 65% vs non-CR to NACT: 57%, *p* = 0.046) 3-yr DFS: 62% (CR to NACT: 64% vs non-CR to NACT: 57%, *p* = 0.03)	NR	NR
Nguyen (2020) [29]	21	GC	CR: 72	3-yr OS: 69% (NACT + TMT: 26.0%)	NR	4 patients (8%) had grade 3+ late toxicity
Royce (2021) [30]	74	NR	NR	5-yr OS: NACT + TMT 32% vs TMT 31% 10-yr OS: TMT 13.3% vs NACT + TMT 13.0%	OS: 308/462 (NACT + TMT) vs 1459/2104 (TMT)	NR
Thompson (2017) [31]	16 (0.8–51)	GC	NR	2-yr OS: NACT + TMT 69% vs TMT 67% (*p* = 0.28) 2-yr DFS: NACT + TMT 81% vs TMT 65% (*p* = 0.6)	NR	Bowel toxicity grade 3+: 18% Urinary toxicity grade 3+: 2 patients

bRFS = bladder recurrence-free survival; CMV = cisplatin, methotrexate, and vinblastine; CR = complete response; CSS = cancer-specific survival; DFS = disease-free survival; GC = gemcitabine and cisplatin; MFS = metastasis-free survival; MVAC = methotrexate, vinblastine, doxorubicin (Adriamycin), and cisplatin; NACT = neoadjuvant chemotherapy; NR = not reported; OS = overall survival; PD = progressive disease; PR = partial response; RFS = recurrence-free survival; RT = radiotherapy; SD = stable disease; TMT = trimodality therapy.

a Event number/total number for each outcome and treatment arm (eg, NACT vs no NACT, CR vs non-CR).

b Not reported separately by arm.

Among nine studies (*n* = 450), the NACT CR rate ranged from 19% to 87% [19], [20], [21], [23], [24], [26], [28], [29], [31]. Case-mix differences appeared to contribute to this variability; for example, Jiang et al [26] reported lower CR rates in an older, more advanced cohort, whereas Cho et al [23] reported higher CR rates despite similar regimens and cycles.

Post-TMT CR rates (reported in six studies, *n* = 791) were 72–86% [19], [20], [24], [27], [28], [29]. Salvage cystectomy rates (reported in ten studies, *n* = 900) ranged from 2% to 22% [10], [19], [20], [21], [22], [23], [24], [26], [29], [31]. Reported toxicities were generally manageable. Gemcitabine-cisplatin was the most common NACT regimen (ten studies, *n* = 1100) [19], [20], [21], [22], [23], [26], [27], [28], [29], [31]. One study incorporated nivolumab with gemcitabine-cisplatin as part of NACT, but the independent effect of immune checkpoint inhibition could not be isolated [23].

### 3.4 Meta-analysis

Although 14 studies met the inclusion criteria, only six studies [10], [20], [21], [23], [27], [30] provided adjusted HRs for oncological outcomes and were included in the meta-analyses.

#### 3.4.1 Effect of NACT use

**Fig. 2:**
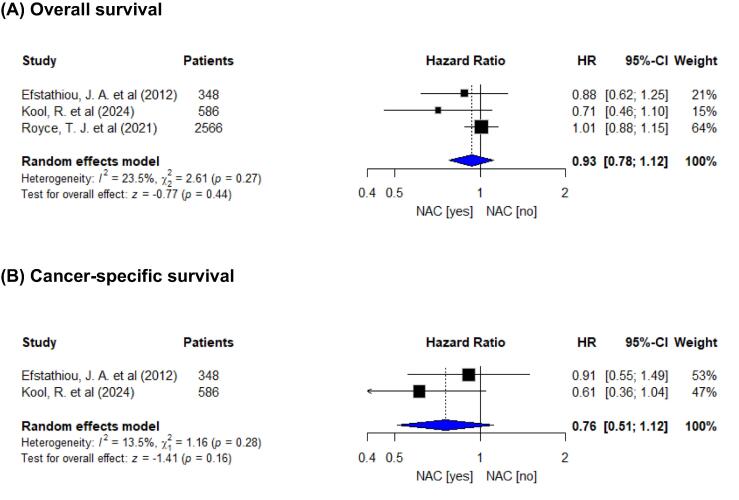
Forest plots for the association between the use of neoadjuvant chemotherapy (NACT) and survival among patients with muscle-invasive bladder cancer treated with trimodality therapy: (A) overall survival and (B) cancer-specific survival. Pooled effects were estimated with a random-effect inverse-variance model using adjusted hazard ratios (HRs) from the most fully adjusted models. Squares are proportional to study weight, horizontal lines indicate 95% confidence intervals (CIs), and diamonds represent pooled HRs. An HR of <1.0 favors NACT. The reported heterogeneity statistics correspond to Cochran’s Q, τ^2^, and I^2^.

Three studies (*n* = 3500) contributed to the NACT versus no NACT analysis for OS [10], [27], [30] and two studies (*n* = 934) for CSS [10], [27]. NACT use was not significantly associated with OS (HR 0.93, 95% CI 0.78–1.12; *p* = 0.40) or CSS (HR 0.76, 95% CI 0.51–1.12; *p* = 0.16; Fig. 2). Heterogeneity was low to moderate and not statistically significant (OS: *Q* = 2.61, *p* = 0.27; I^2^ ≈ 23%; CSS: *Q* = 1.61, *p* = 0.28; I^2^ ≈ 38%). Visual inspection suggested no outlying effects.

#### 3.4.2 Association between response to NACT and outcomes

**Fig. 3:**
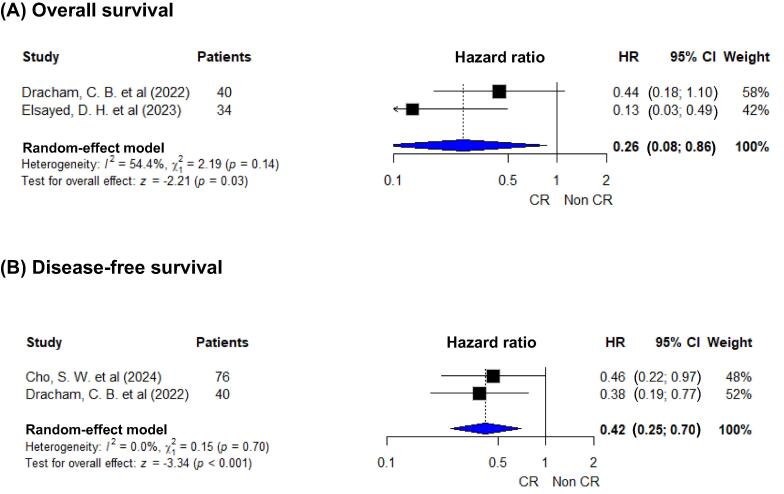
Forest plots for the association between response to NACT and survival among patients receiving TMT: (A) overall survival and (B) disease-free survival. Response is defined as a complete response (CR) versus a non CR (partial response, stable disease, or progression). A random-effect inverse-variance model was used. Symbols represent the same features as those described in Fig. 2. An HR of <1.0 favors a CR. CI = confidence interval; HR = hazard ratio; NACT = neoadjuvant chemotherapy; TMT = trimodality therapy.

Responder analyses were conducted separately from the primary comparisons, as these involved nonoverlapping cohorts consisting only of patients who received NACT. Two studies (*n* = 74) informed the association of an NACT response (CR vs non-CR) with OS [20], [21] and two (*n* = 116) with DFS [20], [23]. A response to NACT was associated with significantly improved outcomes (OS: HR 0.26, 95% CI 0.08–0.86; *p* = 0.03; DFS: HR 0.42, 95% CI 0.25–0.70; *p* < 0.001; Fig. 3). For OS, heterogeneity was not statistically significant (Q = 2.19, *p* = 0.14; I^2^ ≈ 54%); however, the small number of studies precluded sensitivity analyses or formal assessment of small-study effects.

#### 3.4.3 Certainty of evidence

The certainty of evidence was rated as low to very low for all oncological outcomes according to the GRADE framework, primarily due to the observational design, limited numbers of studies contributing adjusted estimates, and the small number of events (Supplementary Table 3).

### 3.5 Sensitivity and additional analyses

Given the small number of eligible and adjusted studies included in each meta-analysis, formal assessments of small-study effects (eg, Egger’s test) and leave-one-out sensitivity analyses were not performed. Prespecified subgroup analyses (by NACT regimen, radiosensitizer, or study period) were not feasible or were underpowered due to inconsistent reporting.

## 4 Discussion

To our knowledge, this is the first systematic review and meta-analysis to evaluate the impact of NACT in the context of TMT for MIBC. Across adjusted nonrandomized studies, we did not observe a survival benefit with routine NACT before TMT in unselected patients. Given the moderate/serious risk of bias in most included studies and heterogeneity in patient selection and protocols, these findings should be interpreted cautiously and are not definitive.

In pooled, adjusted analyses, NACT was not associated with improvements in OS or CSS. Most included evidence was retrospective; although we restricted inclusion to studies with multivariable adjustment to mitigate confounding, residual confounding remains possible. With the exception of Cho et al [23], all studies evaluated conventional cisplatin-based chemotherapy. While cisplatin has long been the standard in MIBC, the treatment landscape is shifting with immunotherapy [32]. The NIAGARA trial reported benefit with perioperative durvalumab plus gemcitabine–cisplatin in the RC setting [33], raising the hypothesis that immune checkpoint inhibitor (ICI)-based neoadjuvant strategies could also add value when integrated with TMT.

CR is an established surrogate for OS in RC cohorts [34]. Consistent with this, our meta-analysis shows that a response to NACT is associated with improved OS and DFS among patients treated with TMT, suggesting that chemosensitivity is prognostic even if routine NACT does not improve population-level outcomes. However, the optimal restaging strategy after NACT to accurately identify complete responders remains uncertain [35]. Importantly, these responder analyses reflect a prognostic association rather than a direct therapeutic benefit of NACT. The observed survival benefit among responders should be interpreted with caution, because these analyses were conducted in distinct, nonoverlapping cohorts consisting only of patients who received NACT. Therefore, these findings do not directly demonstrate a benefit of NACT compared with no NACT. Instead, they may reflect selection factors, with patients who respond well to NACT inherently having more favorable tumor biology. The small numbers of studies and events further limit the certainty of these response-based estimates.

NIAGARA’s high pathological CR rate and our prior meta-analysis in the RC setting—showing a higher pathological CR with ICI chemotherapy versus chemotherapy alone (41% vs 18%; *p* < 0.01)—support further evaluation of ICI-based neoadjuvant regimens within standardized TMT frameworks [1], [33]. Ongoing phase 3 trials (KEYNOTE-992 [36] and INTACT [37]) will help clarify this role. Based on the designs of the KEYNOTE-992 and INTACT trials, which evaluated pembrolizumab and atezolizumab in combination with TMT, it appears that ICIs were administered both concurrently with TMT and as adjuvant therapy. Although ICIs were not incorporated during the NACT setting, the findings from these studies suggest that future treatment protocols may consider the integration of ICIs. Moreover, considering that the AMBASSADOR trial demonstrated that adjuvant pembrolizumab improved DFS in patients with MIBC after radical surgery [38], adjuvant ICI therapy may also further define its role in patients achieving a CR after TMT.

### 4.1 Limitations

First, reliance on prospective and retrospective nonrandomized cohorts introduces risks of selection bias, unmeasured confounding, and differential loss to follow-up. Second, the number of adjusted studies per endpoint was limited, reducing power and precluding robust subgroup analyses. Third, responder analyses involved distinct NACT-only populations, contributing additional uncertainty and preventing direct inference regarding the effect of NACT versus no NACT. Moreover, these analyses were based on small sample sizes and did not incorporate time-dependent methods to mitigate the immortal time bias, further limiting the certainty and generalizability of these findings. Fourth, heterogeneity in NACT regimens, patient selection, and chemoradiotherapy protocols—as well as differences in endpoint definitions and surveillance—limits cross-study comparability. Moreover, patients selected for NACT may have been fitter yet harbored more aggressive disease, creating competing biases difficult to resolve statistically. Finally, our findings do not exclude benefit in specific subgroups; identification of the predictors of a response to NACT in the TMT setting remains a key priority. An NACT response may also inform management: patients achieving a CR could be suitable for bladder preservation, whereas nonresponders might be better served by RC—hypotheses that require prospective validation.

### 4.2 Future perspectives

Future studies should explore the integration of molecular biomarkers to refine patient selection and surveillance within TMT pathways. Circulating tumor DNA (ctDNA) and urinary tumor DNA (utDNA) hold promise as biomarkers for minimal residual disease (MRD) detection and relapse prediction [39], [40]. Powles et al [41] reported that patients who were ctDNA positive experienced improved DFS in the atezolizumab arm versus the observation arm in a randomized phase 3 trial of adjuvant atezolizumab versus observation in operable urothelial carcinoma. In addition, the preliminary results of the TOMBOLA trial (NCT04138628), which assessed ctDNA in MIBC patients undergoing NACT and RC, demonstrated a 98% RFS rate among ctDNA-negative patients [42]. Moreover, utDNA has shown noninvasiveness and superior tumor detection capabilities for early recurrence monitoring in small-scale studies [39]. The use of ctDNA and utDNA analyses could enable molecular risk stratification and MRD detection, allowing intensification for high-risk cases and de-escalation for molecularly disease-free patients. Incorporation of ctDNA/utDNA monitoring into prospective TMT protocols could help personalize therapy and minimize overtreatment.

Additionally, since enfortumab vedotin plus pembrolizumab (EV + P) has rapidly become the new standard of care in the first-line setting for patients with metastatic urothelial carcinoma [43], trials assessing EV + P in the perioperative setting have gained increasing attention. The ongoing KEYNOTE-905/EV-303 [44] and KEYNOTE-B15/EV-304 [45] trials are evaluating perioperative EV + P in the RC setting for MIBC, which may inform future incorporation of this combination into the TMT framework. These developments could enable a more personalized approach to bladder preservation.

## 5 Conclusions

Across adjusted observational evidence, NACT before TMT is not associated with a statistically significant survival benefit in unselected patients with MIBC. These conclusions are based on limited-quality data without dedicated randomized trials. Modern neoadjuvant approaches based on immunotherapy or targeted therapy may prove more effective when embedded in standardized TMT protocols and should be tested prospectively.

  ***Author contributions:*** Shahrokh F. Shariat had full access to all the data in the study and takes responsibility for the integrity of the data and the accuracy of the data analysis.

  *Study concept and design*: Matsukawa, Yanagisawa.

*Acquisition of data*: Miyajima.

*Analysis and interpretation of data*: Miyajima.

*Drafting of the manuscript*: Miyajima, Matsukawa, Miszczyk, Roessler, Inoue, Alqahtani, Alfarhan, Urabe, Mori.

*Critical revision of the manuscript for important intellectual content*: Karakiewicz, Kimura.

*Statistical analysis*: Miyajima.

*Obtaining funding*: None.

*Administrative, technical, or material support*: None.

*Supervision*: Shariat.

*Other*: None.

  ***Financial disclosures:*** Shahrokh F. Shariat certifies that all conflicts of interest, including specific financial interests and relationships and affiliations relevant to the subject matter or materials discussed in the manuscript (eg, employment/affiliation, grants or funding, consultancies, honoraria, stock ownership or options, expert testimony, royalties, or patents filed, received, or pending), are the following: Shahrokh F. Shariat received honoraria from Astellas, AstraZeneca, BMS, Ferring, Ipsen, Janssen, MSD, Olympus, Pfizer, Roche, and Takeda; reports a consulting or an advisory role at Astellas, AstraZeneca, BMS, Ferring, Ipsen, Janssen, MSD, Olympus, Pfizer, Pierre Fabre, Roche, and Takeda; and reports being a member of the speakers’ bureaus of Astellas, AstraZeneca, Bayer, BMS, Ferring, Ipsen, Janssen, MSD, Olympus, Pfizer, Richard Wolf, Roche, and Takeda.

  ***Funding/Support and role of the sponsor:*** None.

  ***Acknowledgments:*** Marcin Miszczyk was supported by the European Urological Scholarship Program (EUSP) Scholarship of the European Association of Urology (EAU).
